# Do Melanocytes Have a Role in Controlling Epidermal Bacterial Colonisation and the Skin Microbiome?

**DOI:** 10.1111/exd.70071

**Published:** 2025-03-06

**Authors:** Omera Bi, David Caballero‐Lima, Stephen Sikkink, Gill Westgate, Sobia Kauser, Jacobo Elies, M. Julie Thornton

**Affiliations:** ^1^ Centre for Skin Sciences, Faculty of Life Science University of Bradford Bradford UK; ^2^ Labskin UK, York Biotech Campus Sand Hutton UK; ^3^ Faculty of Life Sciences University of Bradford Bradford UK

**Keywords:** bacteria, full thickness skin equivalent, immune response, melanocytes, skin microbiome

## Abstract

In addition to producing melanin to protect epidermal keratinocytes against DNA damage, melanocytes may have important roles in strengthening innate immunity against pathogens. We have developed a functional, pigmented, human full‐thickness 3D skin equivalent to determine whether the presence of melanocytes impacts epidermal bacterial growth and regulates the expression of genes involved in the immune response. We introduced primary epidermal melanocytes to construct a 3‐cell full‐thickness skin equivalent with primary dermal fibroblasts and epidermal keratinocytes. Immunohistochemistry verified the appropriate ratio and spatial organisation of melanocytes. Alpha‐MSH induced melanogenesis, confirming an appropriate physiological response. We compared this 3‐cell skin equivalent with the 2‐cell version without melanocytes in response to inoculation with 3 species of bacteria: *Staphylococcus epidermidis, Corynebacterium striatum*, and *Cutibacterium acnes.* There was a significant decrease in the colonisation of bacteria in the skin equivalents containing functional melanocytes. There was increased expression of immune‐response genes (*S100A9, DEFB4A, IL‐4R*) following microorganism exposure; however, there were marked differences between the unpigmented and pigmented skin equivalents. This physiologically relevant human 3D‐skin equivalent opens up new avenues for studying complex skin pigmentation disorders, melanoma, and UV damage, as well as the rapidly evolving field of the skin microbiome and the balance between commensal and pathogenic species.

**Abbreviations**
3D: three dimensional
ALI: Air liquid interface
C. acnes: Cutibacterium acnes
CFU: colony forming units
C. striatum: Corynebacterium striatum
DEFB4A: Homo sapiens defensin beta 4A
IL‐4R: interleukin 4 receptor
MC1R: melanocortin 1 receptor
MITF: Microphthalmia‐associated transcription factor
S. epidermis: Staphylococcus epidermidis
TRP1: Tyrosinase‐related protein 1
α‐MSH: alpha‐Melanocyte Stimulating Hormone

## 1 Background

Understanding human melanocyte biology and pigmentation disorders is hampered by the lack of suitable models; with the diversity of human skin pigmentation adding to its complexity. In addition to producing melanin to protect keratinocytes against DNA damage, it is now believed that melanocytes have important roles in strengthening innate immunity [1, 2, 3]. After the gut, the skin harbours the most abundant microbiome in terms of mass and diversity, with skin commensal bacteria regulating skin homeostasis via innate immunity signalling [4]. While the relationship between melanocytes and skin immunity is acknowledged, there is little understanding of the cross talk between melanocytes and the skin microbiota.

There are no animal models that reflect the diversity of human skin pigmentation or the human skin microbiome. While the culture of primary human epidermal melanocytes is well established [5, 6], 2‐D cultures cannot recapitulate the intimate relationship of surrounding cells, including the microbiota. While human ex vivo explant cultures provide models with melanocytes in their correct spatial environment [7], survival in culture is limited, and the supply of fresh human tissue is restricted. Recently, full‐thickness human skin equivalents have been developed, allowing physiologically relevant investigation of cell‐to‐cell and cell‐to‐matrix interactions [8, 9, 10, 11, 12, 13]. These organoids mimic skin architecture, cellular composition, and functional characteristics, offering a more representative in vitro model.

Until recently, the successful incorporation of melanocytes into a full‐thickness human skin model was limited, but lately several groups have shown significant progress in this area. Such models have demonstrated the correct localisation of melanocytes in the basal layer of the epidermis, mimicking their natural position in human skin [9, 14, 15]. Furthermore, melanogenesis has been demonstrated in response to forskolin [16] and UV stimulation [17].

A commercially available human full‐thickness skin equivalent developed by Labskin features a functional stratified epithelium and is widely recognised for its accurate prediction of skin irritation and toxicity. It is extensively used for safety testing of drugs and cosmetics and can be colonised with skin microflora [18]. Therefore, we have adapted this methodology, incorporating melanocytes to construct a 3‐cell, full‐thickness, pigmented human skin equivalent. The human skin microbiome is dominated by the genera of *Cutibacterium* spp., *Staphylococcus s*pp., and *Corynebacterium* spp. Therefore, we used this advanced 3‐cell model to compare the growth of bacterial species from each genus known to demonstrate microbial –microbial interactions between the skin microbiome and pathogens, namely *Staphylococcus epidermidis, Corynebacterium striatum*, and *Cutibacterium acnes* [19, 20, 21, 22, 23] with the original Labskin 2‐cell, non‐pigmented model. In addition, the expression of *S100A9, DEFB4A*, and *IL‐4*, which have crucial roles in antimicrobial defence, immune regulation, and maintaining skin homeostasis, was compared in the 3‐cell pigmented and the 2‐cell unpigmented skin models following growth of the bacterial species on the epidermis.

## 2 Questions Addressed

1. Can we incorporate primary epidermal melanocytes into a human skin equivalent that can accurately recapitulate their spatial organisation in vivo, and that retains their physiological responses in culture by inducing melanogenesis in response to stimulation with α‐MSH?
2. Can the presence of functional melanocytes in a human full‐thickness skin equivalent modulate the growth of bacteria on the epidermis and regulate the expression of genes linked to the immune response of keratinocytes, thereby revealing a novel role in regulating the skin microbiome?

## 3 Experimental Design

### 3.1 Construction of a Pigmented Full‐Thickness Human Skin Equivalent

**FIGURE 1:**
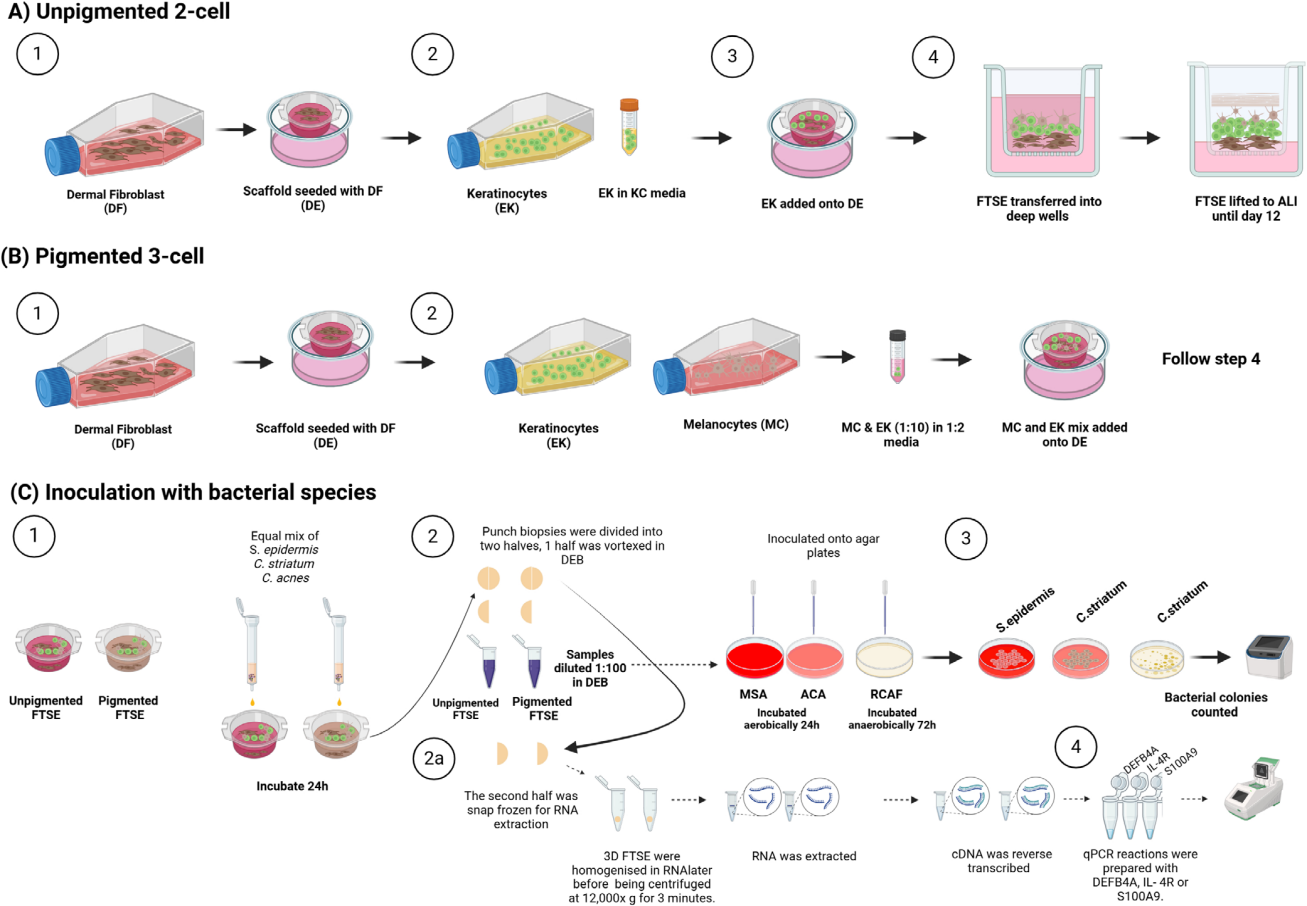
Schematic illustration of the optimised methodology to develop a pigmented 3‐cell human full‐thickness skin equivalent. (A) Unpigmented 2‐cell skin equivalent: (Step 1) A dermal equivalent (DE) was generated with low passage dermal fibroblasts (DF), fibrinogen, and collagen. (Step 2) Once established, early passage epidermal keratinocytes (EK) in keratinocyte media (KC) were seeded on top. (Step 3) Growth media was added to the surface of the full‐thickness skin equivalents (FTSE) and to the bottom of the well every 2 days until the construct was moved to the air– liquid interface (ALI) at day 12. (Step 4) FTSE were transferred into deep wells and growth media was added to the bottom of the well for a further 12 days. (B) Pigmented 3‐cell skin equivalent: The same procedure as described in (A) was followed except at (Step 2) low passage epidermal melanocytes (MC) in melanocyte media were added to the cell suspension of EK in a ratio of 1:10 MC:EK. (C) Inoculation with bacterial species: (Step 1) Pigmented and unpigmented skin equivalents were inoculated with a mixture of *S. epidermidis*, *C. striatum* , and *C. acnes* with a final concentration of each of 1 × 10^4^ CFU cm^2^ before incubating at 37°C with 5% CO^2^ for 24 h. (Step 2) Each FTSE was divided in half and 5 × 8 mm punch biopsies were taken, diluted in 1:100 Dey‐Engley Neutralising Broth (DEB), vortexed, and then plated onto Mannitol Salt Agar (MSA) and Aerobic Coryneform Agar (ACA) for aerobic culture at 37°C for 24 h, and Reinforced Clostridial Agar (RCAF) for anaerobic culture at 37°C for 72 h. (Step 3) Bacterial colonies were counted on each agar type to determine CFU/cm^2^. (Step 2a) Punch biopsies were taken from the 2nd half of each FTSE and stored in RNA later. (Step 4) RT‐qPCR was performed to quantify the expression of S100A9, DEFB4A, and IL‐4A, with gene expression normalised to GAPDH and β‐actin.

Primary human melanocytes were obtained from a juvenile male with moderate pigmentation (PromoCell, Lot 3 071 704) and maintained in Melanocyte Medium 2; primary human epidermal keratinocytes pooled from juvenile foreskin (CellnTec, Lot EI2202108) were maintained in Keratinocyte Media (Scientific Laboratory Supplies, UK) and human dermal fibroblasts from a 55‐year‐old female donor (Gibco, UK, Lot 2 943 323) were maintained in Dulbecco's modified Eagle medium with 10% foetal bovine serum and used at passage 2–4 to generate full‐thickness skin equivalents. The method was adapted from the previously described 2‐cell Labskin skin equivalent [18] (Figure 1). Melanocytes were added to the suspension of keratinocytes at a ratio of 1:10 before adding to the dermal explants, and the constructs were incubated as previously described [18].

### 3.2 Stimulation of Melanogenesis

Alpha‐MSH (α‐MSH) (R&D Systems) was added directly to the epidermis (10^−5^–10^−6^ M) 24 h before the construct was lifted to the air– liquid interface. Thereafter, it was added to the media and refreshed every 2 days.

### 3.3 Identification of Melanocytes by Immunohistochemistry

Skin equivalents were snap frozen in isopentane/liquid nitrogen, embedded in OCT, cut into 6 μm‐thick sections, and stored at–80°C before processing for immunohistochemistry as previously described [24]. The primary antibodies laminin (Abcam, 78 286) and TRP1 (Abcam, 235 447) were both used at a dilution of 1/500. A section of post‐auricular scalp from a 67‐year‐old female was immunostained with TRP1 antibody (1:100 dilution, Santa Cruz Biotechnology, rabbit polyclonal, #sc‐25 543) as previously described [24]. Visualisation was confirmed by diaminobenzidine (DAB) or fluorescence. The number of melanocytes expressing TRP1 was quantitated with ImageJ software. Using a standard area (4 × 1.2 μm) across all images, the number of melanocytes expressing TRP1 was counted. Results are representative of 3 biological repeats; 6 images were used per technical repeat.

### 3.4 Quantification of Gene Expression via RT‐qPCR

RNA was extracted from the full thickness skin equivalent (RNAeasy mini kit, Qiagen), and cDNA was generated using the iScript cDNA Kit (BIO‐RAD, UK). Briefly, the master mix was prepared by combining 4 μL of 5x iScript Reaction mix with 1 μg of RNA template and nuclease‐free water to a total volume of 20 μL. For negative controls, 1 μL of nuclease‐free water was used. Samples were mixed and primed at 25°C for 5 min; cDNA was reverse transcribed at 46°C for 20 min, and reverse transcription was inactivated at 95°C for 1 min using the BIO‐RAD thermocycler c100 (BIO‐RAD, UK). Mastermix qPCR reactions were set up using 10 μL of Applied Biosystems TaqMan Fast Advanced Master Mix, 5 μL of nuclease‐free water, 1 μL of GAPDH‐Cy5 (BioRad, Cat. No. 10031232) 1 μL Beta actin HEX (BioRad, Cat. No. 10031229) 1 μL of TYRP1 FAM (ThermoFisher, Hs00167051_m1) and 2 μL of cDNA. *DEF4B*: Forward Primer: AGACTCAGCTCCTGGTGAAG, Reverse Primer: TTGCGTATCTTTGGACACCA. *S1009*: Forward Primer: AAACACTCTGTGTGGCTCCT, Reverse Primer: TTGGAGGAAGAGACTTTATT. *IL‐4R*: Forward Primer: GCTGCCTGGTCCTGCTGCAG, Reverse Primer: CTGGATGTACATTGGTGTGAAC (Thermofisher, UK). All reactions were carried out in triplicate with an AriaMx Real‐Time PCR System.

### 3.5 Microbe Colonisation of Full Thickness Skin Equivalents

The epidermis of both pigmented and unpigmented skin equivalents was colonised with a combination of *S. epidermidis* (NCTC 11047), *C. striatum (*NCTC 764), or *C. acnes* (NCTC 737) (Sigma‐Aldrich) each at a final concentration of 1 × 10^4^ CFU/cm^2^ and incubated at 37°C in 5% CO_2_ for 24 h before processing for CFU counts. Briefly, 1:100 dilutions of each sample were prepared using Dey‐Engley Neutralising Broth (DEB) before plating onto Mannitol Salt Agar (MSA), Reinforced Clostridial Agar (RCAF) and Aerobic Coryneform Agar (ACA) using a Spiral plater. MSA and ACA plates were incubated aerobically at 37°C ± 2°C for 24 h, and RCAF plates were incubated anaerobically at 37°C ± 2°C for a minimum of 3 days. Bacterial colonies were counted for each agar type to quantitate CFU/cm^2^.

### 3.6 Quantification of Immune‐Response Genes in Response to Bacterial Colonisation

Additional pigmented and unpigmented skin equivalents were colonised with a mixture of the 3 bacterial species, each at a concentration of 1 × 10^4^ and processed for RT‐qPCR (as described above) to quantitate the expression of *S100A9, DEFB4A*, and *IL‐4R*.

## 4 Results

### 4.1 Stimulation of Melanogenesis in a Pigmented Human Full Thickness Skin Equivalent

The Labskin reconstructed unpigmented 2‐cell human full‐thickness skin equivalent was modified to establish a 3‐cell model by incorporating melanocytes, as described in Figure 1.

**FIGURE 2:**
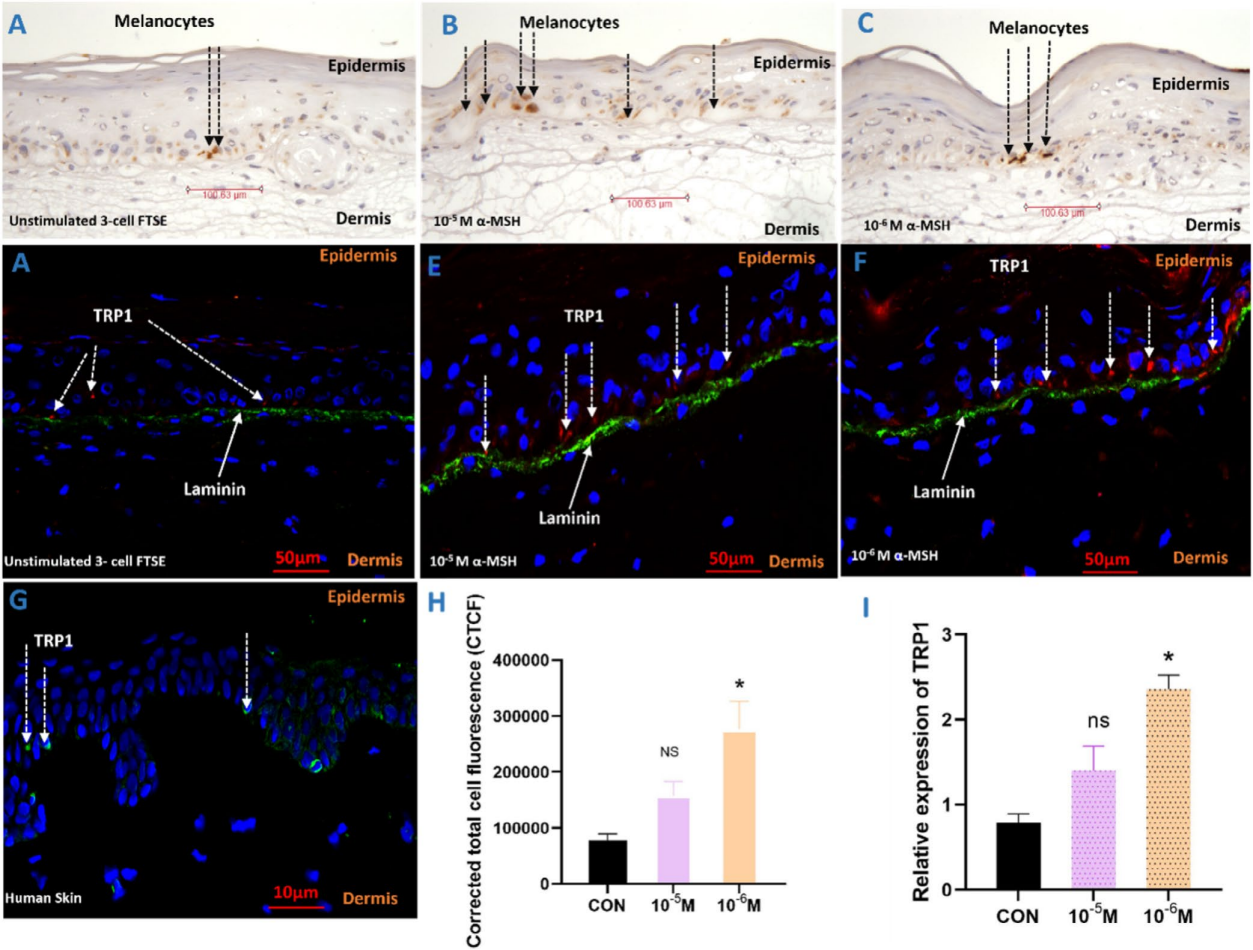
Upregulation of melanogenesis via α‐MSH in a pigmented 3‐cell human full‐thickness skin equivalent. Pigmented 3‐cell full‐thickness skin equivalents were incubated for 12 days in the presence or absence of α‐MSH (10^−5^ or 10^−6^ M), then snap frozen, cut into 6 μM sections and melanocytes localised by immunohistochemistry (peroxidase method) (A–C) or immunofluorescence (D–E). Expression of TRP1 is shown by brown staining (A‐C) or red fluorescence (D–E); basement membrane localised by staining for laminin (green), and nuclei by DAPI (blue). (G) Expression of TRP1 is shown by green fluorescence in human skin in vivo, and nuclei by DAPI (blue). (H) Total cell corrected fluorescence; data are presented as mean +/− SEM of 3 biological repeats (6 images per condition) of the FTSEs. (I) Gene expression of TRP1 in the FTSEs quantitated by RT‐qPCR. Data are presented as mean +/− SEM of 3 biological repeats performed in triplicate. Statistical analysis was performed using one‐way Anova followed by Šídák multiple comparison test (H) and one‐way ANOVA followed by Dunn's multiple comparisons test (I), (ns non‐significant, *p ≤ 0.05).

The melanocyte‐specific marker tyrosinase‐related protein 1 (TRP1) confirmed the spatial orientation of melanocytes in the basal layer of the epidermis (Figure 2A–C) sitting above the basement membrane stained with laminin (Figure 2D–F). Following stimulation with α‐MSH (10^−5^or 10^−6^ M) for 12 days, quantification of TRP1 expression by total cell‐corrected fluorescence demonstrated a significant increase (Figure 2G). Furthermore, gene expression of TRP1 was also significantly increased following stimulation with α‐MSH at both concentrations; gene expression following stimulation with 10^−6^ M α‐MSH was significantly higher when compared to stimulation with 10^−5^ M α‐MSH (Figure 2H).

### 4.2 Altered Skin Microbiota in the Presence of Melanocytes

**FIGURE 3:**
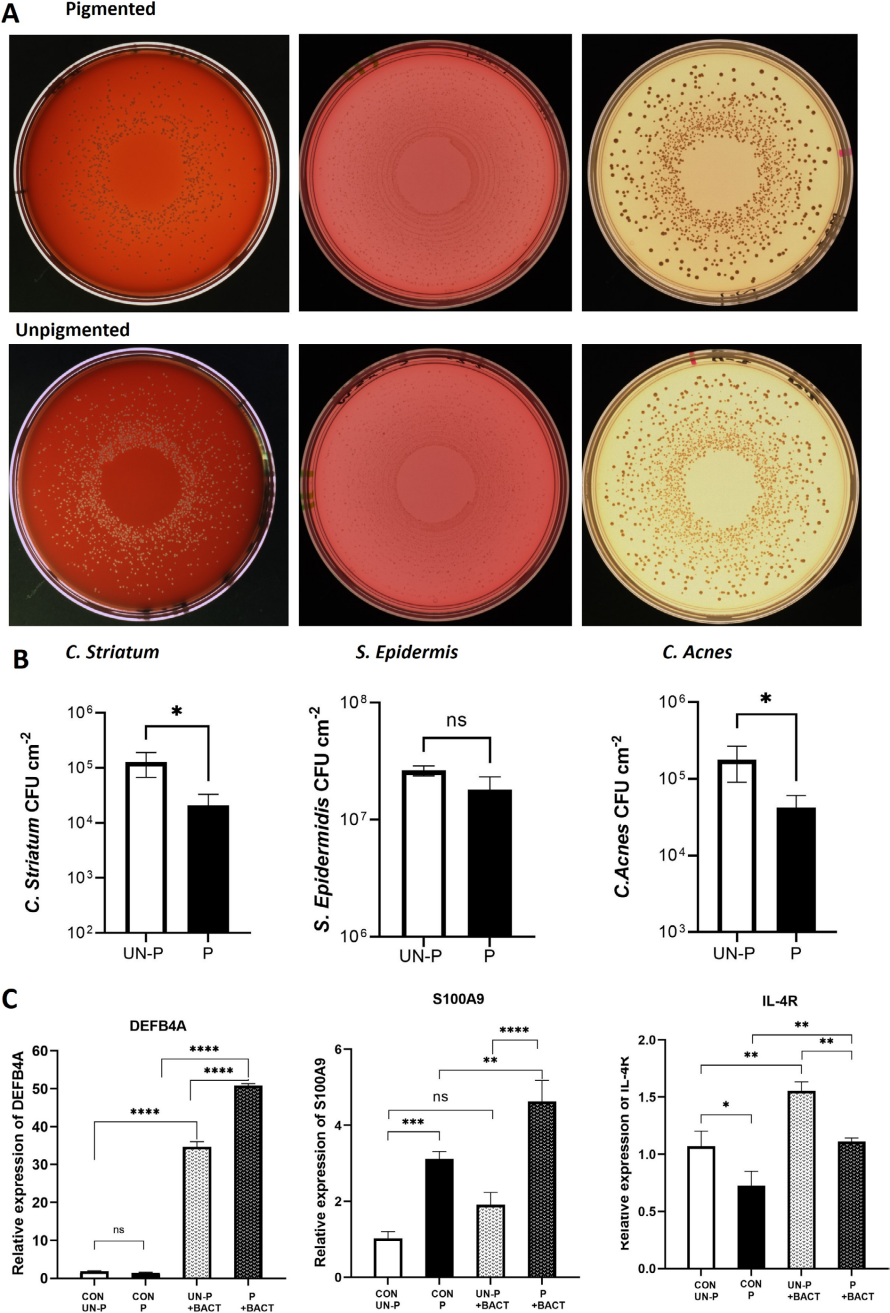
Presence of melanocytes in a human full‐thickness skin equivalent modulates the growth of epidermal microflora and immune‐response genes. A combination of S. *epidermis*, *C. striatum* , or *C. acnes* was grown on the epidermis of unpigmented or pigmented human full‐thickness skin equivalents for 24 h each at a concentration of 1 × 10^4^ CFU cm^2^. Bacterial colonies were counted after plating onto relevant agar to quantitate CFU cm^2^ (A and B). Unpigmented (UN‐P), pigmented (P). Data are presented as mean ± SEM of 5 technical repeats. Statistical analysis was performed using an unpaired two‐tailed t‐test to compare unpigmented and pigmented groups. (C) A combination of S. *epidermis*, *C. striatum* , and *C. acnes* was grown on the epidermis of unpigmented or pigmented human full‐thickness skin equivalents, each at a concentration of 1 × 10^4^ CFU cm^2^ for 24 h. Expression of anti‐microbial genes S*100A9, DEFB4A*, *and IL‐4A* was quantitated by RT‐qPCR following homogenisation of colonised skin equivalents. Gene expression was normalised with GAPDH and β‐actin. Data are representative of three individual skin equivalents and three technical repeats. One‐way ANOVA followed by Dunnett's multiple comparison test was used to assess differences among groups. ns non‐significant, *p* > 0.05, *p ≤ 0.05, **p ≤ 0.01, *** *p* ≤ 0.001, **** *p* ≤ 0.0001. (one‐way ANOVA).

To investigate if the presence of melanocytes in a human full‐thickness skin equivalent can influence the growth of bacteria on the epidermis, the growth of three different species was compared on the 2‐cell (unpigmented) and the 3‐cell (pigmented) human full‐thickness skin equivalents described in Figure 1. The growth of either *S. epidermidis* , *C. striatum* , or *C. acnes* on the epidermis of the full‐thickness skin equivalents was evaluated after 24 h. The number of colonies of *C. striatum* and *C. acnes* on the pigmented full‐thickness skin equivalent was significantly reduced compared to the unpigmented skin equivalent (Figure 3A,B). However, there was no statistical significant difference in the number of colonies of *S. epidermis*.

Further analysis of the full‐thickness skin equivalents identified significant differences in the expression of immune‐response genes quantified by RT‐qPCR between the unpigmented and pigmented full‐thickness skin equivalents after 24 h. This time we inoculated the skin equivalents with a combination of all 3 bacterial species in an equal ratio. Expression of *S100A9*, an alarmin produced by keratinocytes and innate immune cells, was significantly higher in the pigmented skin equivalents under basal conditions and significantly increased following colonisation with bacteria (Figure 3C). Following colonisation, expression was significantly higher in the pigmented skin equivalents compared to the unpigmented ones. While there was no difference in the basal expression of *DEFB4A* between unpigmented and pigmented skin equivalents, colonisation with bacteria significantly increased expression in both models; however, expression was significantly higher in the pigmented skin equivalent in the presence of bacteria (Figure 3C). In contrast, basal gene expression of *IL‐4R* was lower in the pigmented skin equivalents. While bacterial colonisation stimulated expression in both, it remained significantly lower in the pigmented skin equivalent (Figure 3C).

## 5 Conclusion and Perspectives

Recently, there has been an increased focus on improving full‐thickness human skin equivalents from the 2‐cell keratinocyte and dermal fibroblast models by including additional cells such as vascular cells [13], Langerhans cells [17, 25] and melanocytes [9, 16, 17, 26, 27]. Developing reproducible pigmented skin equivalents is a major step forward for studies of pigmentation disorders including vitiligo, melasma, melanoma, and UV‐induced DNA damage. Construction of skin equivalents also allows a better understanding of the diversity of human skin pigmentation by sourcing melanocytes from different genders, skin types (Fitzpatrick scale I–V), anatomical regions, and across the life span.

We have successfully generated a reproducible 3‐cell full‐thickness human skin equivalent, with functional melanocytes inter‐dispersed within basal proliferating keratinocytes sitting on a defined basement membrane above the dermal equivalent (Figure 1). Rather than UV, which induces DNA damage, we used α‐MSH (10^−5^ and 10^−6^ M) to induce melanogenesis, which was validated by an increased expression of TRP1 (Figure 2) confirming activation of the melanocortin 1 receptor (MC1R) and transcriptional regulation of melanogenesis by Microphthalmia‐associated transcription factor (MITF) [28]. While the higher concentration was not as potent, this may be due to increased dendricity or differentiation of melanocytes [29].

While full‐thickness skin equivalents have been widely used for studies of barrier function, we must consider that in vivo human skin is colonised by a distinctive, commensal microbiome, not only important in eliciting defence and immune responses, including protection against pathogens, but also contributing to the differentiation of the epidermis and barrier function [30]. The role of the microbiome in pigmentary disorders is starting to be acknowledged; recent studies have highlighted a role in the aetiology of vitiligo, with a less diverse microbiome accompanied by an increase in specific bacterial taxa that may contribute to its pathogenesis [31]. Furthermore, UV exposure can change the microbial composition, potentially disrupting the balance of commensal bacteria and promoting the growth of pathogenic species; a recent study has highlighted differences in the skin microbiome of melanoma patients, which may be linked to the progression of the disease and response to treatment [32].

Therefore, we compared the growth of three key commensal bacterial species of the skin microbiome in regulating skin homeostasis, that is, *C. acnes*, *S. epidermidis* , and *C. striatum* . We compared their growth on the epidermis of the 3‐cell skin equivalent described in Figure 1, with the 2‐cell skin equivalent that lacks melanocytes. The growth of *C. acnes* and *C. striatum* was significantly lower in the presence of melanocytes, suggesting that melanocytes can control the population of microbes on the upper layer of a stratified epidermis (Figure 3). This is of significance because, while all 3 species are commensal microbes of the healthy skin microbiome, dysbiosis can lead to negative interactions during skin colonisation or infection, and overgrowth/undergrowth of one species may impact other members of the skin microbiota [19]. In contrast, the growth of *S. epidermidis* was similar in both. Recent studies have highlighted the beneficial effects of *S. epidermidis* on skin barrier integrity by increasing ceramide content to prevent dehydration [33]. *S. epidermidis* has evolved mechanisms to sense features of host antimicrobial defence, for example, host antimicrobial peptides (AMPs) [34, 35] enabling it to outcompete virulent pathogens, such as *C. acnes* and *S. aureus* [23]. Therefore, maintaining appropriate levels of *S. epidermidis* will be beneficial for skin barrier integrity.

To investigate potential mechanisms of action, we determined whether immune‐response genes were upregulated. *S100A9* is an alarmin produced by keratinocytes and innate immune cells, and when combined with *S100A8*, it forms calprotectin, which exhibits strong antimicrobial properties and modulates inflammation [36, 37]. Since melanocytes express receptors for calprotectin, they may be activated by keratinocytes in a paracrine manner [38]. Under basal conditions, *S100A9* expression was significantly higher in skin equivalents containing melanocytes, suggesting that their presence may regulate keratinocyte expression. Colonisation with bacteria similarly stimulated expression in both models, confirming an increased proinflammatory response of keratinocytes in the presence of melanocytes (Figure 3C).

Human beta‐defensin‐2 is a potent antimicrobial peptide effective against a broad range of pathogens, with a role in attracting immune cells to infection sites [39, 40]. Under basal conditions, both skin equivalent types expressed low levels of *DEFB4A*, which significantly increased when colonised with bacteria. Interestingly, expression was significantly higher in the pigmented skin equivalents (Figure 3C), suggesting that the presence of melanocytes augments the response.

IL‐4R is the receptor for interleukin‐4 and interleukin‐13, key cytokines in the Th2‐mediated immune response, regulating immune functions, skin barrier integrity, and the skin microbiome [41, 42]. While human keratinocytes constitutively express IL‐4R, in the presence of melanocytes, basal expression was significantly lower in the pigmented skin equivalents (Figure 3C). Since high levels of L‐4 and IL‐13 signalling through IL‐4R impair barrier function [43], this suggests that melanocytes are important in regulating levels for a healthy skin barrier. Colonisation with bacteria stimulated expression to a similar degree relative to basal expression in both pigmented and unpigmented models, again indicating a role for melanocytes in controlling IL‐4/IL‐13 signalling.

While this in vitro model does not fully recapitulate human skin physiology in vivo, with regard to sebaceous lipids, immune cells, vasculature, and a complete microbiome, these data highlight a novel role for melanocytes in controlling bacterial populations resident on the epidermis that may be important in skin barrier function. Furthermore, this 3‐cell, full‐thickness skin equivalent is an excellent model to explore the complex interactions between melanocytes, keratinocytes, dermal fibroblasts, and the skin microbiome, opening up new directions and targets for skin pigmentation as well as the rapidly evolving field of the skin microbiome.

## Author Contributions

O.B. performed the research; G.W., D.C.L., J.E., and M.J.T. designed the research study; D.C.L. contributed essential reagents and tools. O.B., M.J.T., S.S., J.E., D.C.L., and S.K. analysed the data. M.J.T. and O.B. wrote the paper. All authors have read and approved the final manuscript.

## Conflicts of Interest

The authors declare no conflicts of interest.

## Data Availability

The data that support the findings of this study are available on request from the corresponding author. The data are not publicly available due to privacy or ethical restrictions.
